# Unlocking bone repair in osteoporosis by targeting the angiogenic niche

**DOI:** 10.3389/fendo.2026.1790054

**Published:** 2026-04-07

**Authors:** Min Chen, Jing Wang, Jiao Wei, Huirong Feng

**Affiliations:** Department of Upper Limbs, The Affiliated Traditional Chinese Medicine Hospital of Southwest Medical University, Luzhou, Sichuan, China

**Keywords:** angiogenesis, angiogenesis-osteogenic coupling, osteoporosis, therapeutic strategy, VEGF

## Abstract

Osteoporosis (OP) and its related fragility fractures represent a significant global health burden, primarily characterized by reduced bone mass, deteriorated microarchitecture, and compromised self-repair capacity. While current mainstream therapies focusing on anti-resorption and anabolic pathways can slow bone loss, they often fall short in reversing established skeletal deficits and, particularly, in facilitating fracture healing. Growing evidence underscores that angiogenesis is fundamentally coupled with osteogenesis, forming the cornerstone of bone homeostasis and injury repair. However, developing therapeutic strategies targeting this “vascular-bone” axis remains a relatively nascent and underexplored area in the clinical management of both systemic osteoporosis and osteoporotic fractures. This review aims to provide a comprehensive synthesis of dual strategies targeting angiogenesis for the treatment of systemic osteoporosis and the enhancement of osteoporotic fracture healing. We first analyze the pivotal role and regulatory networks of angiogenesis in maintaining bone homeostasis and within the fracture repair microenvironment. Subsequently, we systematically categorize and critically evaluate various intervention strategies based on this approach, including mesenchymal stem cells and their exosomes with paracrine functions, phytochemicals and traditional Chinese medicine compounds possessing dual regulatory activities, non-coding RNAs (miRNAs, lncRNAs, circRNAs) that modulate key signaling pathways, proteins or peptides with defined pro-angiogenic activity, repurposed clinical drugs with potential vascular-modulating properties, and non-invasive physical therapies. The article further compares and contrasts the application of these strategies for systemic bone mass restoration versus localized fracture repair, discussing differences in delivery methods, molecular targets, and expected outcomes. Finally, we address the current challenges in the field—such as target specificity, translational barriers, and the lack of robust clinical evidence—and outline future research directions. By integrating existing knowledge, this review seeks to provide a theoretical foundation and novel perspectives for developing next-generation, synergistic therapies centered on vascular regeneration, designed to concurrently improve bone mineral density and bone quality.

## 1 Introduction

Osteoporosis (OP) is a systemic condition of the skeleton, defined by low bone mass and microarchitectural deterioration, which increases bone fragility and susceptibility to fracture (1). With an estimated 200 million individuals affected globally, OP poses a substantial public health challenge (2). The incidence of osteoporotic fractures, particularly of the hip, spine, and wrist, is projected to climb as populations age, resulting in significant personal suffering and economic costs for healthcare systems worldwide (3).

The development of OP is influenced by a complex constellation of factors, including genetics, hormonal status, environment, and lifestyle (4). Contemporary pharmacological approaches aim to lower fracture risk, maintain bone mineral density (BMD), and support skeletal health (5). Common regimens comprise bisphosphonates, selective estrogen receptor modulators (SERMs), biologic agents like denosumab, and bone-forming drugs such as teriparatide (6). While these treatments are effective, they are not without limitations. Patient adherence is often compromised by real or perceived side effects, complex dosing, and delayed observable benefits. Moreover, drugs may not fully abolish fracture risk in advanced disease or high-risk individuals. Certain therapies carry risks of adverse events, ranging from gastrointestinal discomfort and musculoskeletal pain to rare but serious complications like osteonecrosis of the jaw (7). Consequently, there is a pressing need for personalized treatment strategies that move beyond a universal approach.

The contribution of angiogenesis to bone biology is well-established, yet its therapeutic targeting in OP has been relatively neglected (8). The functional linkage between blood vessel formation (angiogenesis) and bone formation (osteogenesis) is now recognized as a cornerstone of bone integrity and repair (8). This review aims to fill a current knowledge gap by providing a comprehensive overview of angiogenesis-focused management for OP, detailing recent advances and future directions.

## 2 Angiogenesis in OP

Angiogenesis is integral to bone health and is implicated in multiple aspects of OP. New vessel formation ensures the delivery of oxygen and nutrients, enabling the recruitment of osteoprogenitor cells to sites of bone remodeling or injury (9). Therefore, enhancing angiogenic processes can bolster the innate regenerative capacity of bone, potentially reversing OP-related deficits in density and strength (9).

A link exists between chronic inflammation and OP, with angiogenic processes often activated as part of the inflammatory cascade. Pro-inflammatory cytokines can stimulate vessel growth, which may inadvertently amplify osteoclast activity and disrupt osteoblast function, thereby accelerating bone loss (10). Pain is not a direct feature of osteoporosis per se, but often arises following osteoporotic fractures due to local ischemia and inflammation. Improving local blood flow via enhanced angiogenesis may alleviate such fracture-related pain and support functional recovery (11). Delayed fracture union in OP patients is partly attributable to compromised angiogenesis at the injury site. Strategies that boost vessel growth can, therefore, expedite the healing process (12). Furthermore, combining pro-angiogenic adjuvants with standard care may improve the overall therapeutic outcome (13).

In summary, promoting angiogenesis offers a multi-faceted approach to OP management, supporting bone repair, increasing density, relieving pain, accelerating healing, and synergizing with conventional treatments. A deeper understanding of the vascular-bone axis is vital for innovating OP therapeutics.

## 3 Crosstalk between angiogenesis and inflammation in osteoporosis

### 3.1 The inflammatory milieu in osteoporotic bone

Osteoporosis is increasingly recognized as a condition with a significant inflammatory component, particularly in postmenopausal and age-related forms (10). Chronic low-grade inflammation, often termed “inflammaging,” creates a bone microenvironment characterized by elevated levels of pro-inflammatory cytokines, reactive oxygen species, and activated immune cells (9, 10). Within this milieu, angiogenesis—the formation of new blood vessels—is not merely a reparative response but is itself directly regulated by inflammatory signals. While transient, well-orchestrated angiogenesis supports bone repair, persistent inflammatory stimulation often leads to aberrant, non-functional vessel formation that exacerbates bone resorption (11). Understanding the dual role of inflammation in driving both angiogenesis and bone loss is therefore essential for developing vascular-targeted therapies for osteoporosis.

### 3.2 Pro-inflammatory cytokines as regulators of angiogenesis

A network of cytokines dynamically modulates angiogenic processes in the osteoporotic bone niche. The net effect depends on cytokine concentration, duration of exposure, and the cellular context.

TNF-α is a master pro-inflammatory cytokine elevated in the serum and bone marrow of osteoporotic patients (10). It exerts biphasic effects on angiogenesis. At low, acute concentrations, TNF-α stimulates endothelial cell activation and VEGF expression via NF-κB signaling, thereby promoting vessel sprouting (14). However, sustained TNF-α exposure disrupts endothelial integrity, induces apoptosis, and generates immature, leaky vessels that fail to support osteogenesis (10, 14). This duality positions TNF-α as a critical link between inflammatory bone loss and dysfunctional angiogenesis.

IL-1β is another potent osteoclastogenic cytokine that amplifies bone resorption and simultaneously modulates angiogenesis. It upregulates HIF-1α and VEGF in osteoblasts and endothelial cells even under normoxic conditions, thereby enhancing endothelial migration and tube formation (15, 16). Moreover, IL-1β stimulates matrix metalloproteinase (MMP) activity, facilitating vascular invasion into bone remodeling sites. Nevertheless, like TNF-α, chronic IL-1β signaling contributes to pathological vascularization and bone erosion (10).

IL-6, a key mediator of the acute-phase response, is chronically elevated in osteoporosis and correlates with bone loss (10). IL-6 promotes angiogenesis by inducing VEGF and angiopoietin-2 through the JAK/STAT3 pathway (16). This pathway is also exploited by certain pro-angiogenic therapies; for instance, catalpol enhances osteogenesis-angiogenesis coupling via JAK2/STAT3 activation (16). However, persistent IL-6 signaling can destabilize newly formed vessels and shift the bone microenvironment toward chronic inflammation, underscoring the context-dependent role of this cytokine.

IL-17, primarily secreted by Th17 cells, is elevated in postmenopausal osteoporosis and has been implicated in both inflammation and angiogenesis (10). IL-17 stimulates osteoblasts and mesenchymal stem cells to produce VEGF and other angiogenic factors, thereby promoting H-type vessel formation in inflammatory settings. However, IL-17-driven angiogenesis is often coupled with enhanced RANKL expression, tipping the balance toward bone resorption (17).

### 3.3 Pathological consequences of inflammation-driven angiogenesis

In the osteoporotic niche, inflammation-induced angiogenesis frequently results in the formation of structurally abnormal vessels with increased permeability and reduced pericyte coverage (11). These dysfunctional vessels fail to deliver sufficient oxygen and nutrients for bone formation and instead facilitate the infiltration of osteoclast precursors and inflammatory cells. This creates a self-perpetuating vicious cycle: bone resorption releases matrix-bound cytokines, which fuel further inflammation, which in turn stimulates more pathological angiogenesis, ultimately accelerating bone loss (10, 17).

Recent evidence indicates that senescent osteoblasts contribute to this cycle by secreting exosomal miR-139-5p, which induces endothelial cell senescence and impairs angiogenic function, thereby linking cellular aging, inflammation, and vascular dysfunction in osteoporosis (18).

## 4 Angiogenesis-related signaling pathways

The vascular endothelial growth factor (VEGF) signaling axis is a master regulator of blood vessel formation (19). Within bone, VEGF drives the expansion of the vascular network. Its expression level is closely tied to angiogenic activity, influencing bone remodeling dynamics. Disruption of VEGF signaling, whether through excess or deficiency, can skew the balance between bone formation and resorption, contributing to the osteoporotic phenotype (20, 21).

Hypoxic niches, common during bone repair and turnover, activate hypoxia-inducible factor 1α (HIF-1α). This transcription factor coordinates the cellular adaptation to low oxygen, in part by upregulating pro-angiogenic genes like VEGF. In OP, HIF-1α activation may serve as a compensatory mechanism to stimulate vessel growth under stressful conditions (8, 15).

The Notch pathway is a critical modulator of endothelial cell behavior and orchestrates the crosstalk between the vasculature and bone cells. Aberrant Notch signaling can disrupt the precise coordination of osteogenesis and angiogenesis, leading to alterations in bone mass (22–24).

Wnt/β-catenin signaling is a key anabolic pathway in bone, promoting osteoblast differentiation and inhibiting osteoclast formation. It also exerts indirect influence on angiogenesis by regulating the expression of angiogenic factors, including VEGF. Impairment of this pathway is associated with the defective bone formation observed in OP (25–27).

Bone morphogenetic proteins (BMPs) are multifunctional cytokines that coordinate skeletal development and regeneration, with parallel roles in angiogenesis. BMP signaling directs osteoblast lineage commitment and also stimulates endothelial cell functions. Dysregulation of BMP activity has been linked to the pathogenesis of OP and associated vascular deficiencies (28, 29).

These signaling pathways form an interconnected network that synchronizes vessel formation with bone remodeling. Deciphering their intricate interactions is a prerequisite for devising targeted interventions that protect and restore bone through vascular modulation.

## 5 Angiogenesis-osteogenic coupling

**Figure 1:**
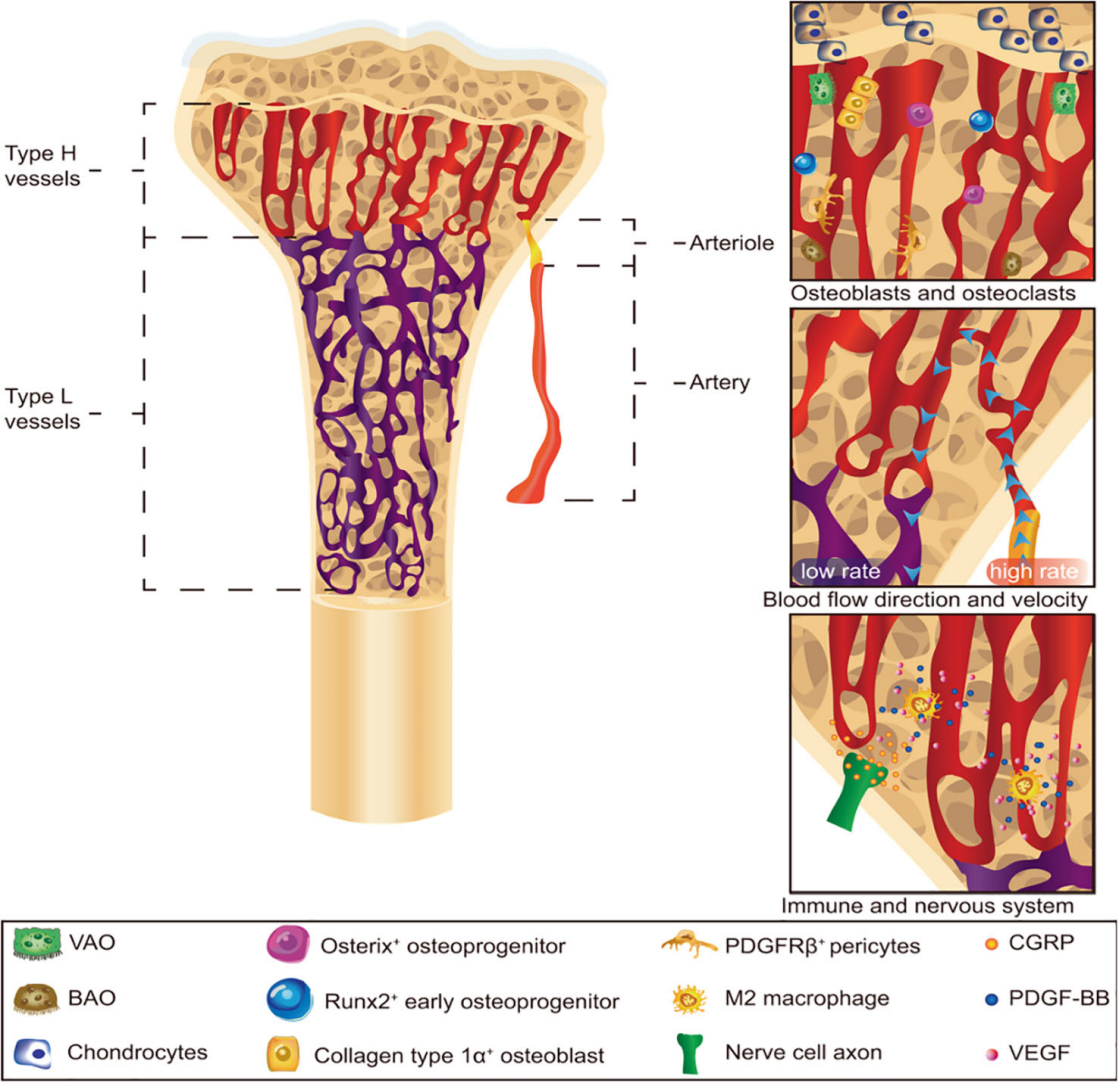
Schematic representation of bone angiogenesis and its coupling with osteogenesis. Reproduced with permission from Ref. (9); Copyright 2023, Wiley Periodicals LLC.

A critical biological concept in bone physiology is the coupled relationship between angiogenesis and osteogenesis (**Figure 1**) (9). The process of bone formation (osteogenesis) is functionally linked to the formation of new capillaries (angiogenesis), with each process supporting the other to ensure skeletal integrity (30, 31).

Osteogenesis is an energetically demanding process. Newly synthesized bone matrix requires a robust blood supply to provide oxygen, nutrients, and growth factors for osteoblasts and other resident cells. The vascular network also facilitates the clearance of metabolic waste products generated during matrix deposition (30–32).

**Figure 2:**
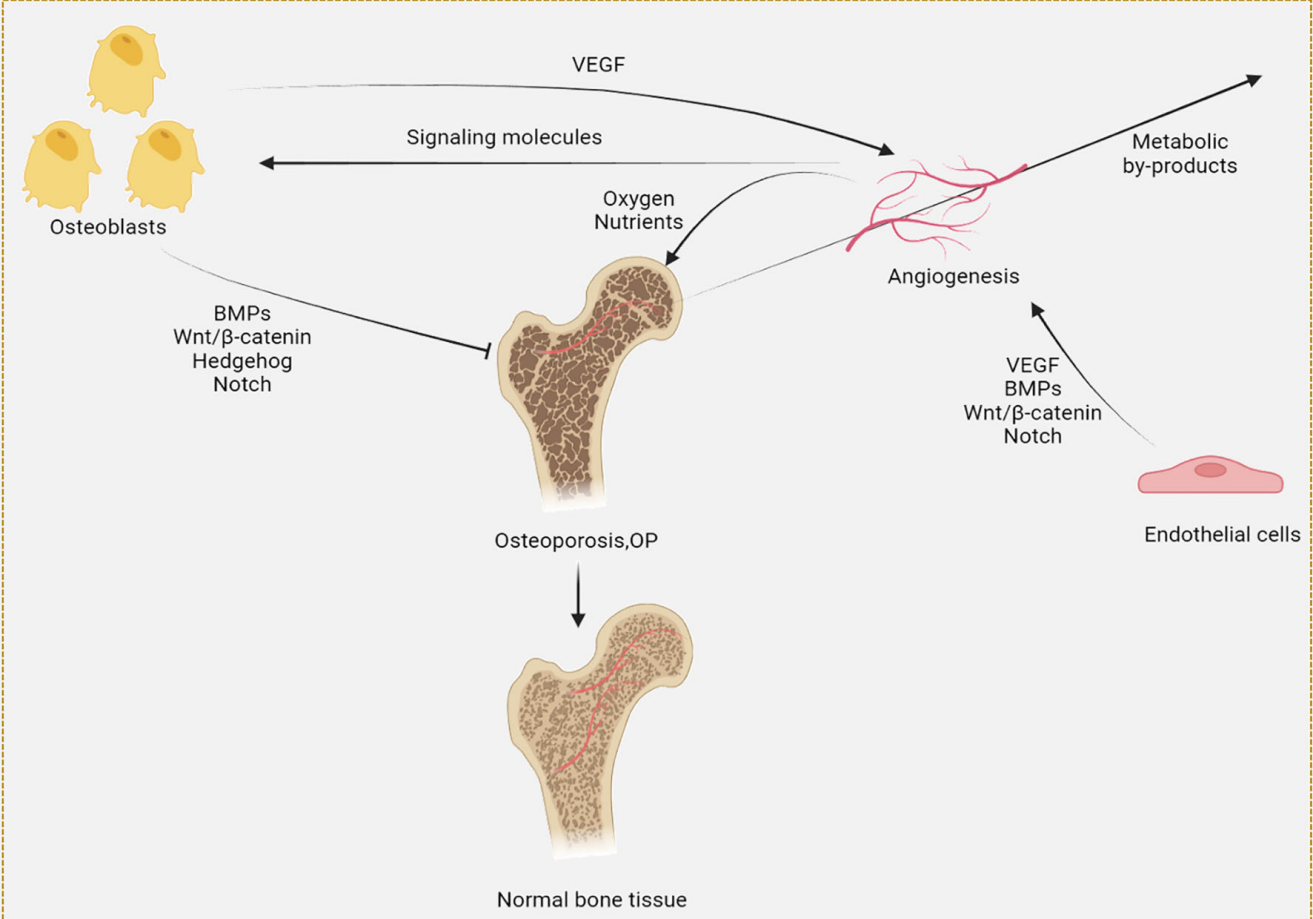
Schematic diagram of the angiogenesis-osteogenesis coupling, which depicts the mutually reinforcing relationship between osteogenesis and angiogenesis in anti-osteoporosis.

Conversely, the bone-forming compartment actively guides angiogenesis. Osteoblasts and osteocytes secrete a panel of pro-angiogenic factors, including vascular endothelial growth factor (VEGF), platelet-derived growth factor-BB (PDGF-BB), and fibroblast growth factor-2 (FGF-2) (33, 34). These factors act on nearby endothelial cells to stimulate their proliferation, migration, and sprouting, thereby extending the vascular network into areas of active bone formation (33, 34). Notably, PDGF-BB derived from osteoblasts not only promotes endothelial cell survival but also recruits pericytes to stabilize nascent vessels, further reinforcing the angiogenic response (35). This reciprocal relationship is thus a self-reinforcing cycle (**Figure 2**). The vasculature provides the necessary infrastructure for bone formation, while the osteogenic compartment directs the patterning and density of the blood supply. This coupling is essential for bone health, and its dysregulation is a hallmark of pathological conditions like OP.

Importantly, the osteoblast-driven angiogenic mechanism is frequently impaired in various disease states. In diabetic osteoporosis, hyperglycemia and advanced glycation end-products suppress VEGF and PDGF-BB expression in osteoblasts, leading to defective vessel recruitment and delayed fracture healing (36, 37). Aging is accompanied by reduced osteoblast number and function, as well as endothelial senescence, which collectively diminish the secretion of and responsiveness to angiogenic cues (38, 39). In chronic kidney disease, accumulation of uremic toxins inhibits osteoblast differentiation and downregulates VEGF, contributing to renal osteodystrophy and poor bone vascularization (40, 41). Avascular necrosis of the femoral head, a condition characterized by irreversible osteocyte death, also involves early impairment of osteoblast-derived angiogenic signals, which exacerbates ischemia and prevents effective repair (42, 43). Thus, preserving or restoring the angiogenic capacity of osteoblasts represents a promising therapeutic strategy for osteoporosis and other skeletal disorders.

In addition to osteoblasts, osteoclasts—particularly pre-osteoclasts—play an active role in bone angiogenesis. It has been demonstrated that pre-osteoclasts secrete high levels of PDGF-BB, which acts on endothelial cells to induce a specialized subtype of CD31^hi^ capillaries (termed type H vessels) (35). These vessels are strongly coupled with osteogenesis, as they are preferentially located in metaphyseal and endosteal regions where active bone formation occurs (30, 35). Type H vessels not only supply oxygen and nutrients but also provide a niche for perivascular osteoprogenitor cells and release factors that promote osteoblast differentiation (24, 44). Conversely, depletion of pre-osteoclasts or targeted deletion of PDGF-BB in these cells leads to a marked reduction in type H vessel density and a concomitant decline in bone mass (35). This discovery highlights the integrated nature of bone remodeling, wherein resorption and formation are not merely balanced but are also coordinated through shared vascular signals. Therefore, therapeutic strategies aimed at enhancing the PDGF-BB/CD31^hi^ vessel axis—either by stimulating pre-osteoclast activity or by directly delivering PDGF-BB—hold considerable promise for reversing bone loss in osteoporosis (13, 35).

## 6 Aging and angiogenesis in OP

Aging is the predominant risk factor for osteoporosis, and its detrimental effects on bone are increasingly attributed to alterations in the skeletal vasculature. With advancing age, the bone microvasculature undergoes progressive regression, characterized by reduced capillary density, impaired endothelial cell function, and diminished regenerative capacity (45). These age-related vascular changes compromise the delivery of oxygen, nutrients, and osteoprogenitor cells, thereby disrupting the delicate equilibrium of bone remodeling and favoring net bone loss (46).

At the molecular level, endothelial cell senescence plays a central role in this vascular deterioration. Senescent endothelial cells acquire a senescence-associated secretory phenotype (SASP), releasing pro-inflammatory cytokines such as IL-6, IL-1β, and TNF-α that create a catabolic microenvironment within bone tissue (47). This not only impairs angiogenesis but also indirectly promotes osteoclastogenesis while suppressing osteoblast function, accelerating age-related bone loss (48).

A particularly relevant discovery in this context is the age-dependent decline of a specific capillary subtype—CD31^hi^ (type H) vessels—which couple angiogenesis with osteogenesis in the murine skeletal system. Kusumbe et al. demonstrated that the abundance of type H endothelium in the metaphysis and endosteum declines steeply with aging, and this reduction precedes and predicts the loss of bone mass (30). This vascular decay is associated with diminished Notch signaling in endothelial cells, further compromising the angiocrine support necessary for perivascular osteoprogenitor maintenance (22).

Notably, restoring vascular function in aged bone has emerged as a tractable therapeutic strategy. Yang et al. showed that transgenic reactivation of the miR-497∼195 cluster specifically in endothelial cells of aged mice rescued type H vessel density and partially reversed age-related bone loss (49). Similarly, pharmacological stabilization of HIF-1α using dimethyloxaloylglycine (DMOG) has been demonstrated to enhance angiogenesis and osteogenesis even in the context of estrogen deficiency-induced bone loss, suggesting that the aged osteoporotic skeleton retains responsiveness to pro-angiogenic cues (50). These findings underscore the importance of considering age-related vascular impairment as both a pathogenic mechanism and a therapeutic target in osteoporosis.

Collectively, aging and angiogenesis are inextricably linked in the pathophysiology of osteoporosis. Therapeutic strategies aimed at promoting bone vascularization must account for the unique challenges posed by the aged endothelial microenvironment. Strategies that rejuvenate the aging endothelium, including senolytic agents, microRNA-based therapies, and endothelial-targeted gene modulation, represent promising frontiers for next-generation osteoporosis treatment (51).

## 7 Treatment strategies based on angiogenesis

### 7.1 Preclinical/investigational drug-based treatment strategies to promote angiogenesis

#### 7.1.1 Mesenchymal stem cells and exosomes

Stem cell-based approaches and exosome therapy represent the vanguard of regenerative medicine. Mesenchymal stem cells (MSCs) are favored for their multipotency, immunomodulatory effects, and relative ease of application (52). Exosomes, nanoscale extracellular vesicles, offer a cell-free alternative with targeted delivery capabilities, low immunogenicity, and high stability, making them attractive therapeutic vehicles (53).

Research indicates that MSCs can counteract postmenopausal OP (PMOP) by revitalizing intraosseous angiogenesis, a process linked to the restoration of bone microcirculation (54). Exosomes from bone marrow MSCs (BMSC-Exos) are internalized by endothelial cells, promoting their proliferation, migration, and tube-forming capacity. This pro-angiogenic effect is mediated, in part, by the transfer of miR-29a from BMSC-Exos to endothelial cells (55). Similarly, exosomes from human induced pluripotent stem cell-derived MSCs (hiPSC-MSC-Exos) enhanced the function of BMSCs from ovariectomized (OVX) rats *in vitro* and stimulated bone regeneration and vessel growth in critical-sized calvarial defects *in vivo* (56).

Treatment of BMSCs with melatonin upregulated markers associated with both bone formation (ALP, OCN, RUNX2, osterix) and vessel formation (VEGF, angiopoietin-2, -4). The hormone was found to augment angiogenesis indirectly by increasing VEGF secretion from BMSCs rather than acting directly on endothelial cells. In OVX rats, melatonin administration enhanced bone repair around defects, correlated with elevated expression of OCN and the vascular markers VEGF and CD31 (57).

The cytokine PDGF-BB, released by osteoclasts, has been identified as a key initiator of a specific subtype of CD31^hi^ vessels that strongly couple angiogenesis to osteogenesis during bone remodeling (35). The pro-angiogenic function of BMSCs is also associated with the abundance of H-type vessels. The histone acetyltransferase GCN5 was shown to promote angiogenesis by enhancing VEGF transcription in BMSCs, and its restoration in OVX mice mitigated femoral vascular loss (58). Mechanical stimulation promotes H-type vessel formation, and this effect is mediated, in part, by the downregulation of exosomal miR-214-3p from BMSCs (59).

Platelet lysates, rich in growth factors, are widely used to support healing. Exosomes derived from these lysates (PL-Exos) improved the osteogenic and angiogenic differentiation of BMSCs and endothelial progenitor cells (EPCs), and enhanced bone formation *in vivo* (60).

#### 7.1.2 Phytochemicals and traditional Chinese medicine formulations

Phytochemicals encompass a diverse array of plant-derived bioactive compounds with significant potential for human health benefit. This category includes flavonoids, polyphenols, alkaloids, and terpenes, frequently associated with antioxidant, anti-inflammatory, and other therapeutic properties (61, 62).

Traditional Chinese Medicine (TCM) formulations consist of complex mixtures of herbs and natural substances, refined through long empirical use to rebalance bodily systems. These prescriptions are often tailored to the individual’s condition according to TCM diagnostic principles. Examples include Xiao Yao San for managing stress and Bu Zhong Yi Qi decoction for boosting vitality and immune function (63, 64). **Table 1** outlines selected phytochemicals and TCM decoctions demonstrating potential in OP treatment via angiogenesis modulation.

**Table 1:** Phytochemicals and traditional Chinese medicine formulations targeting angiogenesis for osteoporosis treatment: experimental evidence and outcomes.

Phytochemical/TCM formula	Category	Source	Experimental system	Administration/dose	Key findings (bone & vessel mprovements)	Proposed mechanism(s)	Ref
Catalpol	Iridoid glycoside	Rehmannia glutinosa	*In vitro*: BMSCs; *In vivo*: OVX rat calvarial defect	*In vitro*: 0.1–10 μM (optimal 1 μM); *In vivo*: Local injection (1 μM, 50 μL) every 2 days for 4 weeks	↑ Bone regeneration (defect closure, BV/TV; quantitative data reported in ref); ↑ Vessel density (CD31^+^ area)	JAK2/STAT3 activation	(16)
Daidzein	Isoflavone phytoestrogen	Soy products	*In vivo*: OVX rat	Oral gavage, 10 mg/kg/day for 12 weeks	Prevented OVX-induced BMD loss; ↑ Type H vessels (CD31^+^Emcn^+^ area)	EGFR/AKT/PI3K cascade	(64)
Icariin	Prenylated flavonol glycoside	Epimedium spp.	*In vitro*: BMSCs, BMEC; *In vivo*: OVX rat calvarial defect	*In vitro*: 10^-6^–10^-4^ M; *In vivo*: CPC scaffold with 10 μM icariin (local); oral 50 mg/kg/day (systemic)	↑ Bone volume (BV/TV); ↑ Vessel number (CD31^+^ vessels)	Promotion of osteogenic/angiogenic differentiation	(65, 66)
Icariin	Prenylated flavonol glycoside	Epimedium spp.	*In vitro*: BMEC	*In vitro*: 10^-5^ M	↑ Extracellular vesicle VEGF and TGF-β1 content; enhanced BMEC viability, migration, and angiogenesis	Modulation of exosomal cargo via VEGF/TGF-β1	(67)
Sesamin	Lignan	Sesame	*In vitro*: ATDC5, HUVEC; *In vivo*: Rat osteoporotic fracture model	*In vitro*: 1–50 μM; *In vivo*: Oral gavage, 20 mg/kg/day for 4 weeks	Accelerated fracture healing (callus volume); ↑ Vascularization (vessel volume)	BMP2 signaling	(68)
Total flavonoids of Rhizoma Drynariae (TFRD)	Flavonoids	Rhizoma Drynariae	*In vivo*: Rat distraction osteogenesis model	Oral gavage, 75 mg/kg/day during distraction	↑ Regenerated bone (BMD, BV/TV); ↑ Type H vessels (CD31^+^Emcn^+^ density)	PDGF-BB/VEGF/RUNX2/OSX axis	(69)
Total flavonoids of Rhizoma Drynariae (TFRD)	Flavonoids	Rhizoma Drynariae	*In vivo*: Rat distraction osteogenesis model	Oral gavage, 75 mg/kg/day during distraction	↑ Type H vessel formation	PDGF-BB/PDGFR-β (not HIF-1α/VEGF)	(70)
Naringin	Flavonoid	Rhizoma Drynariae	*In vitro*: HUVEC; *In vivo*: OVX rat fracture model	*In vitro*: 10–100 μM; *In vivo*: Oral gavage, 100 mg/kg/day for 8 weeks	↑ Fracture healing (callus BMD); ↑ Microvessel density (MVD)	VEGF/VEGFR-2 signaling	(71)
Naringin	Flavonoid	Rhizoma Drynariae	*In vitro*: HUVEC (serum starvation); *In vivo*: OVX rat	*In vitro*: 10–100 μM; *In vivo*: Oral gavage, 100 mg/kg/day for 8 weeks	↓ Endothelial apoptosis; ↑ MVD; ↑ femoral BMD	Anti-apoptosis (ER/mitochondrial pathways)	(72)
Vitexin	Flavonoid	Caella leaves	*In vitro*: HUVEC (hypoxia); *In vivo*: OVX rat	*In vitro*: 5–20 μM; *In vivo*: Oral gavage, 20 mg/kg/day for 12 weeks	↑ Bone mass (femoral BMD); ↑ Angiogenesis (CD31^+^ area); ↑ Endothelial migration and tube formation	VDR/PI3K/AKT/eNOS	(73)
Curcumin	Phenolic compound	Turmeric	*In vitro*: BMSCs (high glucose); *In vivo*: Diabetic osteoporosis mouse	*In vitro*: 1–10 μM; *In vivo*: Intraperitoneal, 50 mg/kg/2 days for 8 weeks	Rescued high-glucose impairment of osteogenesis/angiogenesis; ↑ Type H vessels	NF-κB inhibition	(14)
Panax notoginseng saponins (PNS)	Tetracyclic triterpenoids	Panax notoginseng	*In vivo*: OVX mouse	Oral gavage, 100 mg/kg/day for 12 weeks	Restored OVX-induced bone loss (trabecular BV/TV); ↑ CD31 and OCN expression	Not detailed; promotes angiogenesis-osteogenesis coupling	(74)
Cinnamaldehyde	Aldehyde compound	Cinnamon	*In vivo*: OVX rat calvarial defect (with β-TCP scaffold)	Scaffold loaded with 0.1% (w/w) cinnamaldehyde	↑ Bone formation (new bone area); ↑ Neovascularization (vessel density)	Upregulation of OCN, VEGF, CD31	(75)
Bu-Shen-Tong-Luo Decoction (BSTLD)	TCM formula	Multiple herbs	*In vivo*: OVX rat	Oral gavage, 4.5 g crude drug/kg/day for 12 weeks	↓ Bone resorption (CTSK, TRAP^+^ cells); ↑ Angiogenesis (HIF-1α↑, VEGF↑, CD31^+^ area)	RANKL/OPG modulation; HIF-1α/VEGF	(17)
Modified Qing’e Pills	TCM formula	Multiple herbs	*In vitro*: HUVEC; *In vivo*: Glucocorticoid-induced osteoporosis rat	*In vitro*: 5% medicated serum; *In vivo*: Oral gavage, 1.5 g/kg/day for 8 weeks	Prevented GC-induced bone loss (L4 BMD); ↑ HUVEC proliferation and tube formation; ↑ HIF-1α, VEGF	HIF-1α/VEGF	(76)

BMSC, bone marrow mesenchymal stem cell; BMEC, bone microvascular endothelial cell; HUVEC, human umbilical vein endothelial cell; OVX, ovariectomized; CPC, calcium phosphate cement; BMD, bone mineral density; BV/TV, bone volume/total volume; MVD, microvessel density; CTSK, cathepsin K; TRAP, tartrate-resistant acid phosphatase; GC, glucocorticoid; ER, endoplasmic reticulum; NR, not reported in the cited reference.

↑ stands for increase/rise/upregulation, while ↓ is the opposite.

Catalpol, an iridoid glycoside isolated from Rehmannia glutinosa, possesses a broad spectrum of biological activities including neuroprotection and anti-inflammation. It was shown to stimulate osteoblastic differentiation of BMSCs and foster BMSC-mediated angiogenesis *in vitro* via the JAK2/STAT3 pathway. In an OVX rat model of calvarial defects, catalpol administration enhanced both bone regeneration and associated vascularization (16).

Daidzein, an isoflavone phytoestrogen found abundantly in soy products, demonstrates protective effects against OVX-induced bone loss. Its mechanism involves promoting the formation of type H vessels in cancellous bone, thereby facilitating bone formation, potentially through activating the EGFR/AKT/PI3K cascade (64).

Icariin, a prenylated flavonol glycoside from Epimedium species, is a prominent anti-osteoporotic agent (65). It potentiates the osteogenic and angiogenic differentiation capacity of stem cells in culture. When incorporated into a calcium phosphate cement scaffold and applied to skull defects in OVX rats, icariin significantly improved bone and vessel formation, showing systemic benefits (66). Furthermore, icariin modulates the molecular cargo of extracellular vesicles from bone microvascular endothelial cells (BMEC-EVs), increasing VEGF and TGF-β1 content. These modified BMEC-EVs exhibited enhanced protective effects on endothelial cells, boosting viability, migration, and angiogenic function (67).

Sesamin, a lignan with estrogenic properties, is another regulator of bone metabolism. It activates the BMP2 signaling pathway, inducing chondrogenic and angiogenic responses *in vitro*. In a rodent osteoporotic fracture model, sesamin treatment accelerated the early phase of fracture repair and significantly increased vascularization at the healing site (68).

Total flavonoids extracted from Rhizoma Drynariae were reported to enhance angiogenesis and bone repair in a rat model, upregulating CD31 and activating a signaling cascade involving PDGF-BB, VEGF, RUNX2, and OSX (69). A separate study revealed that these flavonoids specifically drive the formation of type H vessels via the PDGF-BB/PDGFR-β axis, with a higher density of these vessels correlating with superior regenerative outcomes (70).

Naringin, another key flavonoid from Rhizoma Drynariae, aids fracture healing in osteoporosis. It was found to promote angiogenesis in OVX rats by influencing the VEGF/VEGFR-2 signaling pathway (71). Naringin also stimulates the proliferation of vascular endothelial cells in a time- and concentration-dependent manner and protects them from serum starvation-induced apoptosis. Increased microvessel density, positively correlated with bone mineral density, was observed in naringin-treated OVX rats (72).

Vitexin, a flavonoid from Caella leaves, mitigates bone damage and upregulates the expression of osteogenic and angiogenic markers *in vivo*. Its action involves the VDR/PI3K/AKT/eNOS signaling pathway, through which it protects endothelial cells under hypoxia and promotes their migratory and tube-forming abilities *in vitro* (73).

Curcumin, the active component of turmeric, known for its antioxidant and glucose-regulating effects, counteracts the suppressive impact of high glucose on BMSCs’ osteogenic and angiogenic potential. It does so by inhibiting the NF-κB pathway activation under hyperglycemic conditions. In a mouse model of diabetic osteoporosis, curcumin treatment reduced bone loss and encouraged the formation of type H vessels (14).

Panax notoginseng saponins (PNS), derived from Panax notoginseng, are recognized for improving vascular circulation. PNS administration restored bone mass in OVX mice, accompanied by increased levels of the vascular marker CD31 and the osteogenic marker OCN in bone tissue, indicating simultaneous activation of angiogenesis and osteogenesis (74).

Cinnamaldehyde, a bioactive compound from cinnamon, which has known osteoblast-stimulating effects, promoted new bone mineralization in OVX rats. It upregulated the expression of OCN, VEGF, and CD31, and was associated with increased neovascularization at the defect site (75).

Bu-Shen-Tong-Luo Decoction (BSTLD), a TCM formulation used for PMOP, suppressed bone resorption in OVX rats, as evidenced by reduced expression of calcitonin receptor and cathepsin K (CTSK). It also promoted angiogenesis by stabilizing HIF-1α and increasing VEGF expression, while modulating the RANKL/OPG axis (17).

The classic TCM formula Qing’e Pill, modified for enhanced efficacy, prevented glucocorticoid-induced osteoporosis. It functioned by upregulating HIF-1α and VEGF expression. *In vitro* studies confirmed that Modified Qing’e Pills boost vascular endothelial cell proliferation and protect against glucocorticoid-induced vascular damage (76).

#### 7.1.3 LncRNA/MiRNA/CircRNA

Long non-coding RNAs (lncRNAs) are transcripts longer than 200 nucleotides that lack protein-coding potential but play significant regulatory roles. They influence gene expression, chromatin state, and epigenetic regulation by interacting with DNA, RNA, and proteins, and are implicated in both normal physiology and diseases like cancer and neurodegeneration (77, 78).

The lncRNA TCONS-00023297 was demonstrated to regulate the balance between osteogenic and adipogenic differentiation in human BMSCs, and to coordinate the osteogenesis-angiogenesis interplay via the miR-608/RUNX2/SHH axis (79). In OP patients, reduced expression of lncRNA GAS5 was found to enhance angiogenesis by acting as a sponge for miR-10a-3p, leading to increased VEGFA expression (80). Furthermore, lncRNA SNHG1 was upregulated in OVX mice, where it directly inhibited miR-181c-5p and activated Wnt/β-catenin signaling via SFRP1. SNHG1 knockdown promoted osteogenic differentiation of BMSCs, whereas its overexpression favored osteoclastogenesis and impaired angiogenesis (81).

MicroRNAs (miRNAs) are short non-coding RNAs, approximately 22 nucleotides long, that fine-tune gene expression post-transcriptionally, typically by binding to the 3’ UTR of target mRNAs, leading to translational repression or mRNA decay. They are crucial regulators of development, differentiation, and metabolism, and their dysregulation is a feature of many diseases, making them attractive therapeutic targets (82).

Exosomal miR-139-5p, derived from senescent osteoblasts, was shown to impair endothelial cell function. This miRNA, elevated in senescent cells and their exosomes, induced senescence and apoptosis in vascular endothelial cells and suppressed their proliferation and migration by targeting TBX1 (18). miRNA-210 has emerged as a key player in bone vascular pathology. It protects against estrogen deficiency-induced osteoporosis by upregulating VEGF and promoting osteoblast differentiation. 17β-estradiol was shown to induce HIF-1α and VEGF expression in osteoblasts in a dose- and time-dependent manner, a process involving miR-210 (83). Another miRNA, miR-143, promotes osteoblast formation and angiogenesis by directly targeting and suppressing HDAC7, and HDAC7 knockdown rescues the effects of miR-143 inhibition (84).

The miR-497∼195 cluster is highly expressed in CD31^hi^ endothelial cells (ECs), and its expression declines with age. Silencing this cluster in ECs reduced CD31 levels, vascularity, and bone mass. Conversely, transgenic overexpression in mouse ECs attenuated age-related vascular and bone loss. Targeted delivery of agomiR-195 to ECs in aged mice specifically activated angiogenesis and bone formation (49).

Circular RNAs (circRNAs) are stable, covalently closed loop RNAs that arise from back-splicing. They can function as miRNA sponges, protein decoys, and transcriptional regulators, and are involved in various cellular processes and diseases. Their stability and specificity make them promising diagnostic and therapeutic agents (85). CircRNA 0006215 was reported to promote BMSC osteogenesis and enhance osteogenesis-angiogenesis coupling by competitively binding miR-942-5p, thereby increasing RUNX2 and VEGF expression (86).

#### 7.1.4 Proteins

Proteins constitute the primary executers of cellular signaling, and several have been identified as pivotal nodes in the angiogenesis-osteogenesis coupling network. For instance, Osteogenic Growth Peptide (OGP) not only promotes MSC osteogenesis but also upregulates angiogenic factors including VEGF and Fbln5 during differentiation (87). MMP2 Inhibitor 1 (MMP2-I1) exerts dual benefits by activating p38/MAPK in BMSCs for osteogenesis and HIF-1α in HUVECs for angiogenesis, concurrently promoting H-type vessel formation and mitigating OVX-induced bone loss (88). Endothelial ZEB1 maintains Notch signaling via epigenetic regulation; its conditional deletion in ECs impairs bone angiogenesis and subsequent osteogenesis (89). Additionally, the HIF-1α stabilizer DMOG has been repurposed to activate Wnt/β-catenin signaling, enhancing trabecular bone mass and vascularization in osteoporotic models (50). Given the vast landscape of protein regulators in bone angiogenesis—ranging from transcription factors to matrix remodelers—a comprehensive discussion of their mechanistic networks is beyond the scope of this therapeutic-focused review and has been expertly covered elsewhere (13, 35). The examples herein serve to illustrate the druggable potential of protein nodes within the angiogenic-osteogenic cascade.

### 7.2 Clinically approved drug-based treatment strategies to promote angiogenesis in OP models

Parathyroid hormone (PTH), a key regulator of calcium and phosphate metabolism, elevates blood calcium levels (90, 91). Studies on PTH in OP revealed that the fragment PTH (1-34) stimulates new bone formation, angiogenesis, and implant integration in aged rats during early healing phases. It induces an early angiogenic response by secreting factors that recruit BMSCs and enhance osteoclast participation in remodeling (92). Another investigation showed that either PTH1-34 or MSC treatment improved bone healing and callus vascularization. PTH1-34 specifically increased the density of small-diameter vessels (≤50μm), while MSCs promoted larger vessels (>50μm). PTH1-34 treatment elevated CD31 expression in callus and trabecular bone, and PTH1-34-primed MSCs showed increased levels of CD31, VEGFR, VEGFR2, and vWF, indicating a heightened pro-angiogenic state (93).

Dexmedetomidine (Dex), a highly selective α2 adrenergic receptor agonist with sedative and anxiolytic properties (94), promotes osteogenesis and angiogenesis by downregulating miR-361-5p, leading to increased VEGFA expression. miR-361-5p is upregulated in PMOP patients and rat models, suggesting its diagnostic utility. Dex treatment in osteoporotic rats corrected the expression of miR-361-5p and VEGFA, improved BMD-related parameters, and elevated osteogenesis-angiogenesis gene expression in BMSCs (95).

Deferoxamine, a siderophore from Streptomyces pilosus used for iron overload, was evaluated in an osteoporotic bone defect model. Comprehensive analyses, including histology and bone histomorphometry, demonstrated that deferoxamine significantly enhanced bone formation and angiogenic responses in OVX rats (96).

Melatonin, an amine hormone produced by the pineal gland that regulates sleep cycles (97), enhances both osteogenic and angiogenic activities in BMSCs *in vitro*. Treatment increased expression of osteogenic markers (ALP, OCN, RUNX2, osterix) and angiogenic factors (VEGF, angiopoietin-2, -4). Its pro-angiogenic effect is mediated indirectly via VEGF upregulation in BMSCs. In OVX rats, melatonin supplementation boosted the levels of OCN, VEGF, and CD31 during bone defect repair (57).

### 7.3 Drugs in clinical trials targeting angiogenesis for osteoporosis or related indications

Several agents currently in clinical trials—either approved or under investigation for osteoporosis or related bone disorders—exert angiogenic effects through modulation of vascular-bone coupling (**Table 2**). Romosozumab, an anti-sclerostin monoclonal antibody approved for postmenopausal osteoporosis, activates Wnt/β-catenin signaling and has been reported to enhance intraosseous vascularization in preclinical models, contributing to its bone-forming efficacy (98, 99). BPS804, another anti-sclerostin antibody, has completed phase II trials for hypophosphatasia and osteogenesis imperfecta, with potential implications for osteoporosis therapy via angiogenesis-osteogenesis coupling (100). AMG 780, a monoclonal antibody targeting angiopoietin-1 and -2, has undergone phase I evaluation in oncology and represents an emerging approach to modulate vascular stability in bone (101). Moreover, although VEGF inhibitors are widely used in cancer treatment, controlled local delivery of VEGF or HIF-1α stabilizers (e.g., DMOG, roxadustat) is being explored in phase II trials for fracture healing and bone regeneration, highlighting a paradigm shift toward context-dependent pro-angiogenic therapy (102).

**Table 2:** Clinically approved drugs with reported pro-angiogenic activity and their known or potential effects on osteoporosis.

Drug name	Patient population (sex)	Effect on angiogenesis (yes/no/known/unknown)	Effect on osteoporosis (yes/no/known/unknown)	Ref
Atorvastatin	Mixed (M/F)	Yes (promotes VEGF, endothelial function)	Yes (BMD increase in some studies)	(103, 104)
Simvastatin	Mixed (M/F)	Yes (angiogenesis in ischemic models)	Known (osteogenic effects *in vitro*/*in vivo*)	(105, 106)
Erythropoietin (EPO)	Mixed (M/F)	Yes (endothelial progenitor cell mobilization)	Known (bone formation in animal models)	(107, 108)
Enalapril	Mixed (M/F)	Yes (improves microvascular density)	Unknown (limited data)	(109, 110)
Sildenafil	Male predominant	Yes (cGMP-mediated angiogenesis)	Unknown (no clinical data in OP)	(111, 112)
Rosiglitazone	Mixed (M/F)	Yes (pro-angiogenic in some contexts)	No (associated with bone loss)	(113)
Deferoxamine	Mixed (M/F)	Yes (HIF-1α stabilization)	Yes (preclinical OP models)	(42, 96)
Melatonin	Mixed (M/F)	Yes (VEGF upregulation)	Yes (anti-osteoporotic effects)	(57, 114)
Dexmedetomidine	Mixed (M/F)	Yes (via miR-361-5p/VEGFA)	Yes (preclinical evidence)	(95, 115)

### 7.4 Non-invasive physical therapies

In recent years, non-invasive physical modalities have garnered increasing attention for their ability to stimulate angiogenesis and bone regeneration without the need for pharmacological or surgical interventions. Among these, extracorporeal shockwave therapy (ESWT) and low-intensity pulsed ultrasound (LIPUS) have shown particular promise in preclinical and clinical studies of osteoporosis and fracture healing.

ESWT delivers acoustic pressure waves to targeted skeletal tissues, inducing mechanical stimulation that triggers a cascade of biological responses. Multiple studies have demonstrated that ESWT promotes angiogenesis by upregulating pro-angiogenic factors such as VEGF, eNOS, and HIF-1α, while also enhancing endothelial cell proliferation and tube formation (116, 117). In osteoporotic bone defect models, ESWT has been shown to improve vascularization, accelerate bone remodeling, and enhance osteogenic differentiation of BMSCs (118, 119). Notably, ESWT has also been reported to promote chondrogenesis and endochondral ossification, processes closely linked to angiogenesis-dependent bone repair (120). These findings suggest that ESWT may be particularly beneficial for osteoporotic patients who are concurrently receiving angiogenesis-inhibiting drugs (e.g., vismodegib for cancer treatment), as it could locally counteract vascular suppression and support bone healing.

LIPUS is an FDA-approved non-invasive modality primarily used to promote fracture healing. Emerging evidence indicates that LIPUS also exerts pro-angiogenic effects. *In vitro* studies have shown that LIPUS stimulation increases VEGF secretion in osteoblasts and endothelial cells, activates the PI3K/Akt and ERK signaling pathways, and enhances endothelial tube formation (121, 122). *In vivo*, LIPUS treatment has been associated with increased CD31-positive vessel density and improved blood perfusion at fracture sites in osteoporotic animal models (123). These effects are partly mediated by the upregulation of HIF-1α and downstream angiogenic gene expression under mechanical stimulation (124).

Together, these non-invasive physical therapies offer translational advantages, including low systemic toxicity, repeatability, and compatibility with existing pharmacological regimens. Future research should focus on optimizing treatment parameters and evaluating their long-term efficacy in osteoporotic populations.

## 8 Discussion

Current pharmacological interventions for osteoporosis—primarily antiresorptive and anabolic agents—have demonstrated efficacy in reducing fracture risk and preserving bone mineral density. Yet their limitations are increasingly recognized: suboptimal long-term adherence, delayed observable benefits, high costs that exacerbate health disparities, and a non-negligible residual fracture risk, particularly in patients with severe disease or multiple comorbidities (7). These constraints underscore an urgent need for therapeutic paradigms that extend beyond the conventional osteoclast- versus osteoblast-centric framework.

Angiogenesis has emerged as a compelling target in this regard. The discovery that SLIT3, an axon guidance protein, potently stimulates intraosseous vessel growth and that its genetic deletion in mice precipitates bone loss has reframed the vasculature as an active, druggable component of bone homeostasis (13). Indeed, the functional coupling of angiogenesis and osteogenesis is now recognized as a cornerstone of skeletal integrity, and its dysregulation is a hallmark of osteoporosis (8, 30, 31). Pro-angiogenic strategies therefore hold promise not merely as adjuncts, but as a distinct mechanistic avenue to restore bone mass and quality.

This promise is further accentuated by the fact that osteoporosis frequently coexists with—and is aggravated by—pathological conditions inherently characterized by impaired angiogenesis. In diabetes mellitus, chronic hyperglycemia and advanced glycation end-products compromise endothelial function and reduce VEGF bioavailability, leading to defective intraosseous vascularization and delayed fracture healing (36, 37). Encouragingly, pro-angiogenic interventions such as curcumin, miR-210 agomir, or HIF-1α stabilizers have been shown to restore type H vessel formation and improve bone microarchitecture in diabetic osteoporotic animals (14, 50, 83). Ageing, another major risk factor, is accompanied by a progressive decline in CD31^hi^ vessel density and endothelial Notch activity, both directly linked to age-related bone loss (30, 49). Notably, endothelial-specific overexpression of the miR-497∼195 cluster or targeted delivery of agomiR-195 partially reversed vascular and skeletal deficits in aged mice, suggesting that rejuvenating the aged angiogenic niche is a viable anti-osteoporosis strategy (49). In chronic kidney disease, uremic toxins and disrupted mineral metabolism impair HIF-1α signaling and suppress bone angiogenesis, compounding the skeletal fragility of renal osteodystrophy (40, 41); preclinical studies indicate that agents such as deferoxamine can mitigate this deficit (42, 96). Avascular necrosis of the femoral head represents a localized form of ischemic bone failure that often precedes secondary osteoporosis, and its pathogenesis is rooted in subchondral angiogenic collapse. VEGF delivery, stem cells, or exosome-based therapies have been demonstrated to enhance revascularization and prevent osteonecrosis progression in steroid-induced models (42, 43, 125). Collectively, these observations transform the concept of angiogenesis promotion from a theoretical benefit to a clinically relevant necessity in large and underserved patient populations.

**Figure 3:**
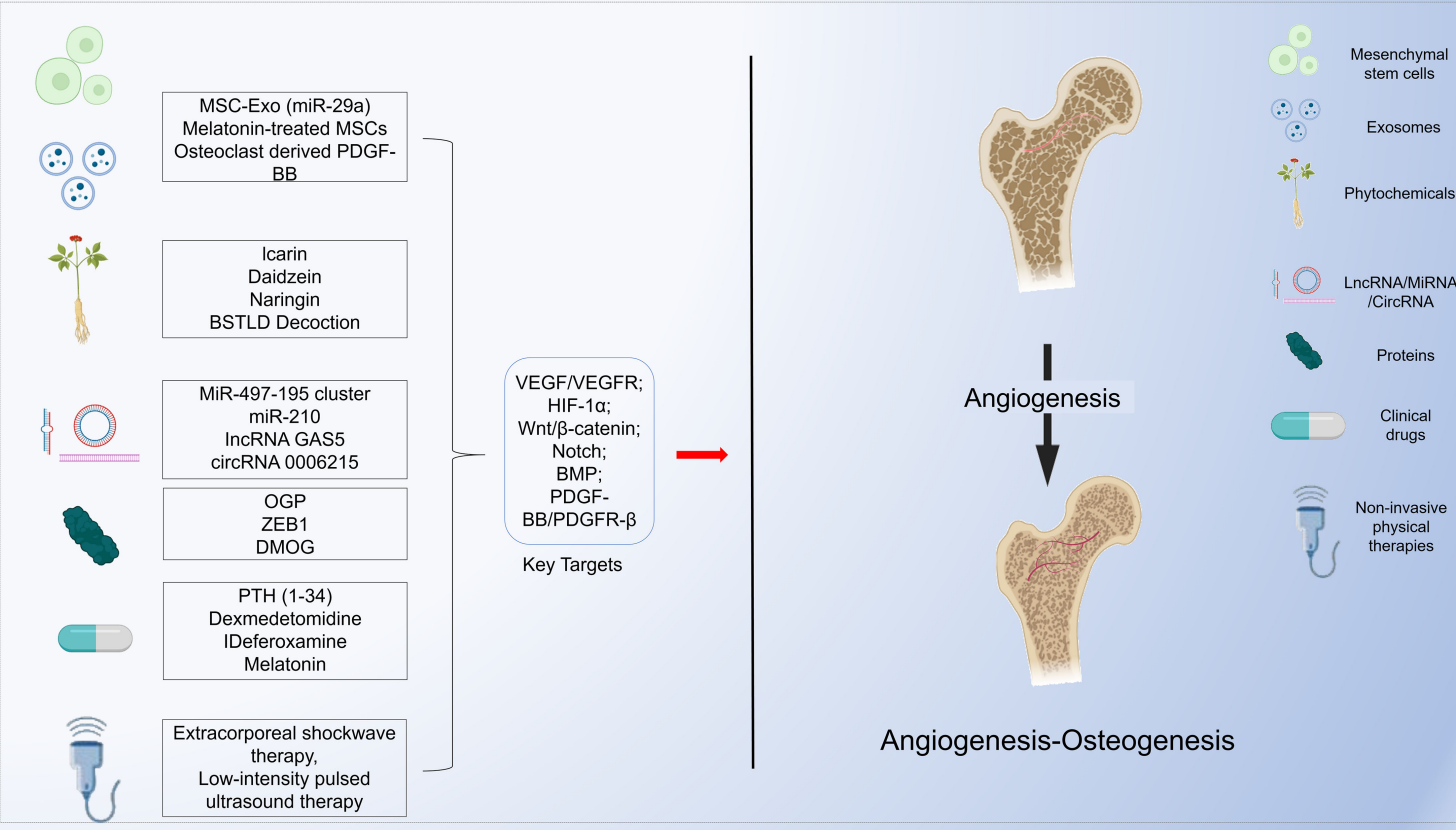
Potential therapeutic strategies for regulating angiogenesis-osteogenesis coupling include mesenchymal stem cells and their derived exosomes, phytochemicals and active ingredients from traditional Chinese medicine, non-coding RNAs (miRNA, lncRNA, circRNA), proteins, repurposed clinical drugs with potential vascular regulatory properties, and non-invasive physical therapies.

Recognizing this translational imperative, a surge of studies has explored pro-angiogenic interventions from diverse angles. As summarized in **Figure 3**, current investigational strategies include mesenchymal stem cells and their exosomes, phytochemicals and traditional Chinese medicine formulas, non-coding RNAs (miRNA, lncRNA, circRNA), recombinant proteins, repurposed clinical drugs, and non-invasive physical therapies. Each of these categories has yielded encouraging proof-of-concept data in preclinical osteoporosis models, validating the feasibility of pharmacologically enhancing bone vascularization.

Nevertheless, substantial obstacles remain before these approaches can be translated into clinical practice. Stem cell- and exosome-based therapies, while biologically potent, face unresolved questions regarding optimal cell source, manufacturing standardization, delivery route, and long-term safety (126). Phytochemicals and herbal decoctions offer advantages in terms of accessibility and multimodal mechanisms, but their complex composition hinders the identification of active principles, and robust clinical trial data are almost entirely lacking. The involvement of miRNAs, lncRNAs, circRNAs, and regulatory proteins in angiogenesis-osteogenesis coupling reveals promising mechanistic nodes, yet their pleiotropic nature raises legitimate concerns about target specificity and off-target effects; moreover, ethical and safety barriers continue to impede the clinical translation of gene-level therapeutics (127–130). Collaboration among researchers, healthcare practitioners, and regulatory bodies will be pivotal in unlocking the complete potential of these groundbreaking therapies to enhance patient outcomes and propel the progress of regenerative medicine. For instance, the Guidelines for Stem Cell Research and Clinical Translation developed by the International Society for Stem Cell Research (ISSCR) represent a successful model of tripartite collaboration among academia, industry, and regulatory bodies (e.g., FDA, EMA), establishing a framework for standardizing and safely evaluating stem cell-based therapies.

Thus, while the field has made remarkable strides in delineating the vascular component of osteoporosis and its therapeutic potential, a substantial gap persists between preclinical discovery and bedside application. Closing this gap will require not only continued mechanistic dissection and technological refinement, but also a deliberate expansion of research focus to encompass the complex comorbid contexts in which osteoporosis most severely afflicts patients.

Noteworthy, it is equally important to recognize that pharmacological inhibition of angiogenesis—a cornerstone strategy in oncology and ophthalmology—can inadvertently compromise skeletal health and precipitate or exacerbate osteoporosis. Vascular endothelial growth factor (VEGF) signaling inhibitors, including the monoclonal antibody bevacizumab and tyrosine kinase inhibitors such as sunitinib and sorafenib, disrupt the tightly coupled angiogenesis-osteogenesis process; clinical and preclinical evidence indicates that these agents suppress osteoblast activity, alter the RANKL/OPG balance in favor of osteoclastogenesis, and significantly increase fracture risk (131, 132). Glucocorticoids, while not classic angiogenesis inhibitors, exert potent anti-angiogenic effects by downregulating VEGF expression and impairing endothelial cell survival and function, a mechanism that contributes substantially to the pathogenesis of glucocorticoid-induced osteoporosis independent of their direct actions on bone cells (133). Similarly, methotrexate has been shown to inhibit endothelial proliferation and delay fracture healing through impaired neovascularization (134). Aromatase inhibitors, used in hormone receptor-positive breast cancer, induce profound estrogen deficiency; given that estrogen physiologically promotes VEGF expression and endothelial nitric oxide synthase activity, these agents indirectly suppress bone angiogenesis and accelerate bone loss, particularly in postmenopausal women (135). These observations carry important clinical implications: patients receiving long-term anti-angiogenic therapy—especially those with pre-existing low bone mass or additional risk factors—should undergo routine bone health monitoring and may benefit from prophylactic interventions such as calcium, vitamin D, or anti-resorptive agents. Moreover, the skeletal toxicity of these drugs provides compelling evidence from the opposite direction for the centrality of the vascular niche in bone homeostasis and reinforces the rationale for developing vascular-targeted osteoanabolic therapies as a next-generation approach to osteoporosis management.

In summary, this review consolidates current knowledge on angiogenesis-centered anti-osteoporosis strategies and underscores their relevance beyond uncomplicated, experimental osteoporosis. By integrating the emerging evidence from both primary disease models and comorbid conditions characterized by angiopathic bone loss, we hope to provide a conceptual foundation for next-generation therapies that leverage vascular regulation to restore skeletal health. It is our conviction that targeting the angiogenic niche is no longer an option, but an imperative—and one that is increasingly within reach.
